# Urinary Microbiota of Healthy Prepubescent Girls and Boys—A Pilot Study

**DOI:** 10.3390/children12010040

**Published:** 2024-12-29

**Authors:** Yulia L. Naboka, Mikhail I. Kogan, Johannes M. Mayr, Irina A. Gudima, Elizaveta M. Koliva, Violetta M. Kotieva, Marina L. Chernytskaya, Vladimir V. Sizonov

**Affiliations:** 1 Department of Microbiology and Virology No. 1, Rostov State Medical University, 344022 Rostov-on-Don, Russia; naboka_yul@rostgmu.ru (Y.L.N.);; 2 Division of Pediatric Urology of the Department of Urology and Human Reproductive Health, Rostov State Medical University, 344022 Rostov-on-Don, Russia; 3 Children’s Day Hospital Liestal, and University of Basel, 4001 Basel, Switzerland; 4 Division of Uroandrology, Regional Children’s Clinical Hospital, 664022 Rostov-on-Don, Russia

**Keywords:** child, healthy, microbial community, microbiota, urine

## Abstract

Background: The urinary microbiota of healthy children has rarely been studied, and potential differences between boys and girls have not been addressed. Thus, this study aimed to compare the urinary microbiota of healthy prepubescent girls and boys. Methods: We included healthy children aged between 4 and 10 years who were free of functional or organic urinary tract diseases and had no history of urinary tract infection. We collected the mean portion of morning urine during natural micturition and determined aerobic and anaerobic microbiota using HiCrome™ chromogenic growth media. We identified microorganisms on the basis of morphotinctural properties and analyzed α- and β-diversity of microorganisms isolated from the urine of boys and girls. Results: Mean age of the children was 6.1 ± 3.2 years. In general, four-component (28.1%) as well as two-component (15.6%), three-component (15.6%), and six-component (12.5%) combinations of microorganisms prevailed in the urine of children. The urine of boys exhibited four-component combinations significantly more often than that of girls (*p* ˂ 0.05), while the urine of girls contained seven-component microbial combinations significantly more often than that of boys (*p* ˂ 0.05). Comparison of multicomponent combinations of microorganisms in boys and girls revealed an overrepresentation of *Enterococcus* spp. in girls (*p* < 0.05). Furthermore, there was a trend towards higher microbial α-diversity in the urine of girls, but the difference between girls and boys was not significant. Conclusions: The urine of healthy prepubescent children contained various aerobic–anaerobic combinations of microorganisms. Their diversity in the urine of girls and boys did not differ significantly. However, the level of α-diversity of microorganisms was higher in girls than in boys. We noted differences in the prevalence of certain taxa of microorganisms in the urine of boys and girls. Our study showed a close functional relationship between aerobic and anaerobic microorganisms detected in the urine of children in more than half of the cases.

## 1. Introduction

In the past, the urinary tract was considered sterile in the absence of symptoms and signs of urinary tract infection (UTI). However, with the development of modern methods of identifying microorganisms, new insight into the role of the microbiota and microbiome of the urinary tract has emerged, both in healthy people and in patients with various urological diseases [1,2,3,4,5].

In adults, the urinary microbiota in the bladder has a number of important functions in analogy with the microbiota of other organs. In particular, it supports the integrity of the uroepithelium and immune defense system of the mucosa thus helping to prevent the development of UTI [6,7,8,9]. Studies of the urinary microbiota and/or microbiome in healthy children of different age groups are largely missing. Kassiri et al. [9] assessed the urobiome in boys (n = 20) aged 3 months to 8 years (mean age 15 months) before initiating routine urological procedures. Various species of microorganisms, such as *Staphylococci*, *Streptococci*, *Propionibacteria*, *Corynebacteria*, *Varibacula*, *Anaerococci*, *Peptoniphili*, and *Serratia*, were found in the boys’ urine collected by catheter [9]. Kinneman et al. analyzed catheter urine samples in infants who suffered from fever and suspected UTI and compared the α-diversity of microorganisms in urine between children who had received antibiotic treatment and those who had not [10]. 

In children with UTIs of various localizations, *Enterobacteriaceae* appear to predominate as the causative bacteria [11,12,13]. Accurate diagnosis of urological diseases in children depends on fully understanding the urinary microbiota in healthy individuals. Thus, this pilot study aimed to characterize the urinary microbiota of healthy prepubescent children and assess any possible differences between boys and girls.

## 2. Materials and Methods

### 2.1. Study Design and Inclusion/Exclusion Criteria

For this prospective cohort study, we included 32 healthy prepubescent children (18 boys, 14 girls) aged between 4 and 10 years (mean age 6.1 ± 3.2 years). Healthiness was defined by the absence of any functional or organic diseases, lack of drug intake (including antimicrobial drugs) for at least 3 months before study onset, and no history of UTIs or other infectious diseases. For eligibility, a written informed consent form signed by the child’s parents or caregiver was required.

Children with a history of acute or recurrent UTI, daytime urinary incontinence and/or nocturnal enuresis, or intestinal dysfunction (diarrhea, constipation) were not eligible for the study.

### 2.2. Ethical Approval

The study was approved by the local independent Ethics Committee of the Rostov State Medical University (protocol number 2/2023, 17 January 2023).

### 2.3. Collection of Urine Samples and Bacterial Analyses

During normal micturition, we collected a single midstream sample (10 mL) of the morning urine into a sterile plastic container (Steril Unicol, HiMedia, Kennett Square, PA, USA) labeled with the child’s identification number. For urine collection, we observed the routine hygiene precautions. Transfer of the samples to the analytical laboratory took less than 30 min.

We analyzed the bacterial composition of the urine samples in accordance with the clinical recommendations termed “Bacteriological urine analysis” (2014) [14]. In addition to the recommended growth media, we used HiCrome™ chromogenic growth media for aerobic and anaerobic microbiota (i.e., MacConkey Agar, HiCrome™ Klebsiella Selective Agar Base, HiCrome Candida Differential Agar, HiCrome™ Enterococci Agar, HiCrome™ Aureus Agar Base, Blood Agar Base, Streptococcus Selection Agar, Rogosa SL Agar, Bifidobacterium Agar, MRS Agar, Anaerobic Agar, Shaedler Agar, and Bacteroides Bile Esculinum Agar), HiMedia Laboratories Private Limited, Mumbai, Maharashtra, India. We employed AnaeroHiGasPack to create anaerobic conditions. Urinary microorganisms were identified by determining morphotinctural properties (a set of dyes for differential staining according to Gram Stains-Kit, HiMedia) and employing cultural and biochemical tests (ENTEROtest, STAPHYtest, ANAEROtests, Erba Lachema, s.r.o., Brno, Czech Republic).

### 2.4. Bioinformatic Processing and Statistical Analysis

We determined α-diversity using the following variables [15]: number of observed species, Shannon index, Simpson index, and Pielou’s evenness index. We assessed these variables separately for boys and girls. All quantitative variables were tested for normal distribution. Most data including Shannon index variables were normally distributed, while the Simpson index and Pielou’s evenness index were not. For normally distributed variables, the means were compared using Welch’s *t*-test. For variables not normally distributed, we used a nonparametric Mann–Whitney test. Because of the small sample size in this study, we used a Shapiro–Wilk test to decide whether the data fitted a normal distribution.

To determine β-diversity of microorganisms isolated from urine of boys and girls, we used principal component analysis and hierarchical cluster analysis. For the latter, we applied the binary classification method that uses the square of the Euclidean distance. Differences of bacterial composition in the urine of boys compared to that of girls were additionally presented in a heat map showing the relative proportion of bacterial strains.

We applied descriptive statistics for the rank variables of microbial concentration levels (i.e., minimum and maximum values, median and quartiles, and mean and standard deviation if applicable). For quantitative variables, we computed the median (Med) and interquartile range with 25th and 75th percentiles. A nonparametric Mann–Whitney test was used to compare the groups. Due to the small number of observations, the exact values of the significance levels were calculated using the Monte Carlo method (99% significance level; 10,000 samples). We used contingency tables to analyze the detection frequencies of microorganisms. Groups were compared using the chi-square test, and exact values of significance levels were calculated using the Monte Carlo method. To determine the relationships between the detection frequencies of the various microorganisms, we computed Pearson contingency coefficients. Significance was verified using the chi-square test with exact values of significance levels computed by the Monte Carlo method. We used statistical package SPSS version 26 (IBM Corp., Armonk, NY, USA) for all analyses.

## 3. Results

**Figure 1:**
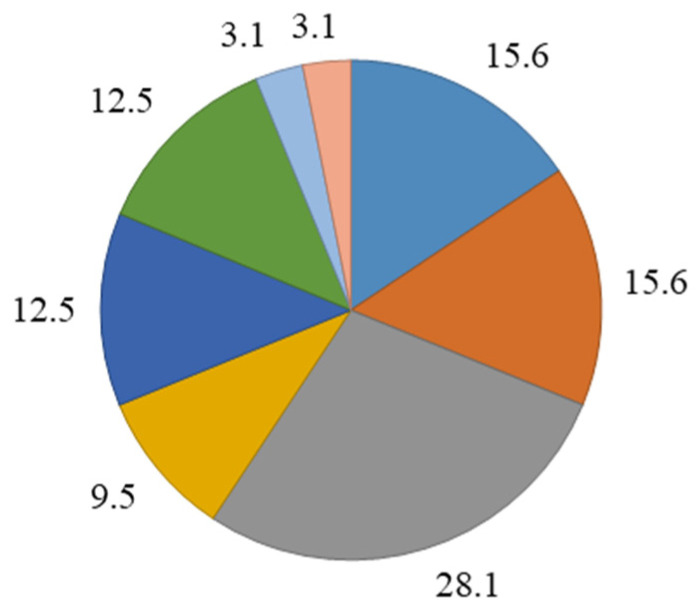
Frequency (%) of multicomponent microbial combinations in the urine of children.

The mean age of the children was 6.1 ± 3.2 years. The urine of all children exhibited various bacterial combinations ranging from two-component to ten-component combinations (Figure 1).

**Figure 2:**
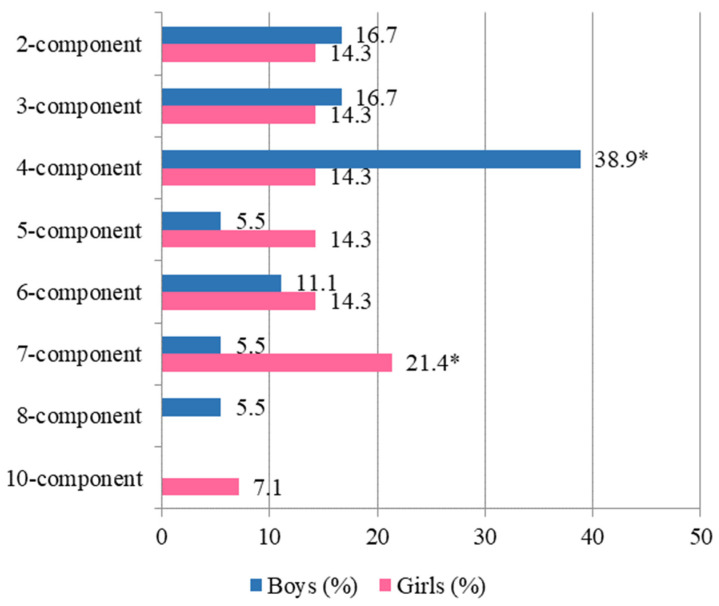
Frequency (%) of multicomponent microbial combinations in the urine of boys and girls (n = 32) * *p* ˂ 0.05.

In general, four-component (28.1%) as well as two-, three-, and six-component combinations of microorganisms prevailed in the urine of children. Boys exhibited four-component combinations significantly more often than girls (*p* ˂ 0.05), while the urine of girls contained seven-component microbial combinations significantly more often than that of boys (*p* ˂ 0.05; Figure 2).

Table 1 shows the main characteristics of the urinary microbiota in boys and girls (n = 32).

In the cluster of aerobic bacteria, *Coagulase-negative Staphylococcus (CoNS)* predominated in the urine of boys (72.2%), while *Enterococcus* spp. prevailed in the urine of girls (64.3%). In the non-clostridial anaerobic bacteria cluster in boys, *Peptococcus* spp. was most prevalent (61.1%), whereas *Eubacterium* spp. was predominant in girls (78.6%). Significant differences in detection frequencies of urinary microorganisms occurred only for undifferentiated *Enterococcus* which occurred more often in girls than boys (*p* = 0.040). The urine of boys contained neither *C. tropicalis* nor *C. krusei,* and the urine of girls contained no *E. faecium* or any of the three types of non-clostridial anaerobic bacteria (*Veillonella* spp., *Fusobacterium* spp., *Prevotella* spp.).

The mean level of bacteriuria in the majority of children was 10^2^ CFU/mL, with the exception of *C. tropicalis*, *C. krusei* (10^3^ CFU/mL), and *Lactobacillus* spp. (10^4^ CFU/mL) isolated from the urine of girls.

α-Diversity (Table 2) of urinary microorganisms was higher in girls than boys, but the difference was not statistically significant.

**Figure 3:**
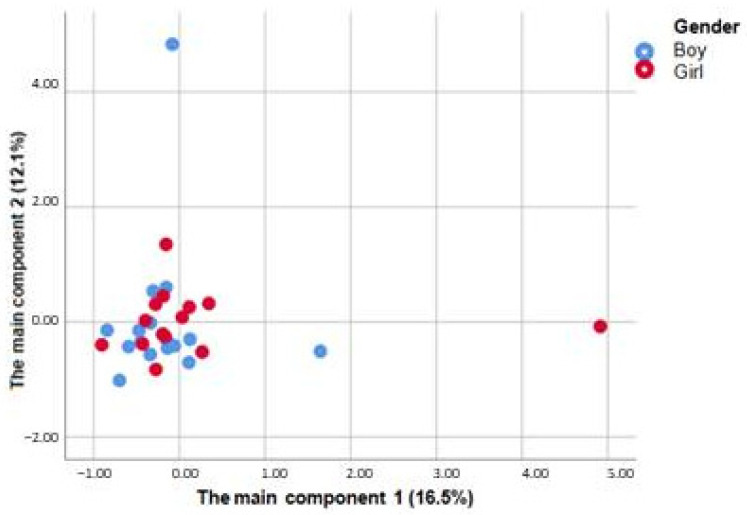
Scatter diagram of the two main components (n = 32).

When comparing the microbial patterns in girls and boys (β-diversity), factor analysis of the main components with varimax rotation revealed 10 main factors that collectively explained 82.2% of the variation of microorganisms. Figure 3 shows the scatter diagram of the two main components, the first of which explained 16.5% and the second 12.1% of the variation of microorganisms. Although there was a slight tendency of an increased first component in girls and increased second component in boys, it was not possible to detect any significant differences in the microbiota of boys and girls in general.

**Figure 4:**
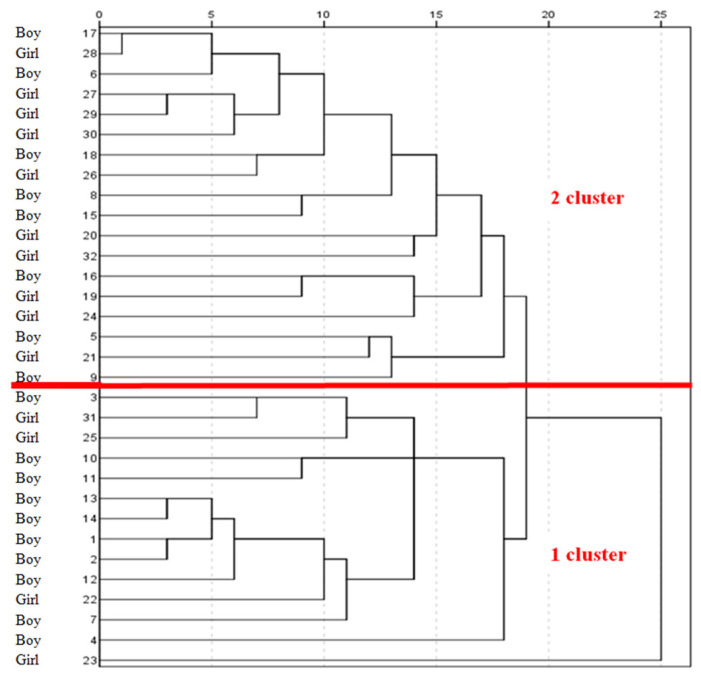
Hierarchical clustering of urinary microbiota of healthy boys and girls (n = 32). The red line indicates the division into two clusters.

We obtained a dendrogram to visualize the two clusters by hierarchical cluster analysis (Figure 4). While boys predominated in the first cluster (71.4%), girls predominated in the second cluster (55.6%), but the difference was not statistically significant (Table 3).

**Figure 5:**
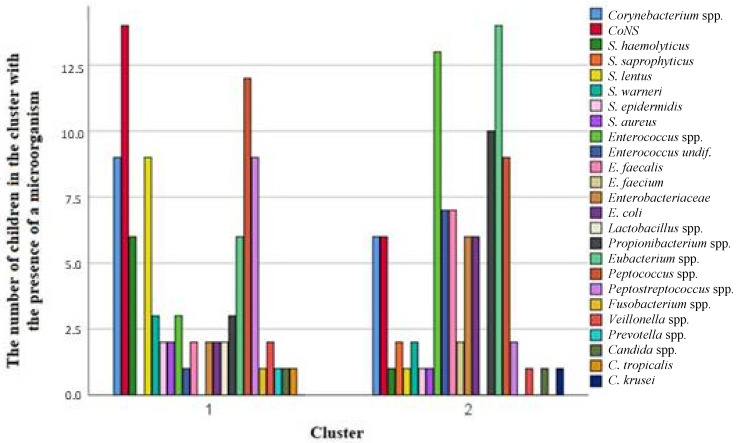
Microorganisms contained in clusters 1 and 2 by number of children.

Figure 5 shows the various microorganisms contained in clusters 1 and 2 by number of children. In the first cluster with an overrepresentation of boys, *CoNS*, *Peptococcus* spp., *Corynebacterium* spp., and *Peptostreptococcus* spp. were detected in the urine of the majority of children. The second cluster consisted of more girls than boys, and *Enterococcus* spp., *Eubacterium* spp., and *Propionibacterium* spp. were the predominant bacteria. This finding corresponds to the gender distribution of microorganisms shown by the heat map (Table 4).

Analysis of the microbial diversity in the urine of children did not reveal any statistically significant differences between the sexes. At the same time, we detected somewhat greater α-diversity of microorganisms in girls, as well as differences in the type of microorganisms prevailing in girls and boys.

Table 5 shows the statistically significant relationships between the detection rate of microorganisms in the urine of boys and girls. In total, 31 significant Pearson contingency coefficients (C*_p_*) were found in the microbiome analysis of the urine of children, of which 18 were detected in boys and 13 in girls. Moreover, a reliable functional relationship (C*_p_*) between aerobic and anaerobic microorganisms was observed in 54.8% of children.

## 4. Discussion

The urinary tract in humans used to be considered sterile, but in recent decades, this hypothesis was refuted [2,16]. In particular, new diagnostic methods such as enhanced quantitative urine culture (EQUC), polymerase chain reaction (PCR), whole genome sequencing (WGS), and next-generation sequencing (NGS) including sequencing of the 16S-rRNA gene brought to light previously undetected microorganisms and a peculiar urinary microbiome [3,6,16,17].

The origin of the microbiota and/or microbiome of the urinary tract is still not entirely understood. In newborns, the composition and function of the urinary microbiome depend on the type of delivery at birth, the mother’s microbiota, antibiotic exposure, and type of feeding [6,10,18]. The main source of urinary microbiota in infants is assumed to be the microbial communities of the mother’s gastrointestinal tract and vagina. The infant’s microbiota is formed and stabilized 2 to 3 years after birth [6,18,19]. In adolescents, pubertal hormonal changes may affect maturation of the urinary tract microbiome [6,11]. However, the effect of age on the urinary tract microbiota of healthy children has rarely been analyzed previously [6].

Our study employed established bacteriological methods, albeit using an expanded set of nutrient media with aerobic and anaerobic cultivation techniques. Therefore, our results cannot be compared directly with those obtained by sequencing the various regions of the 16S-rRNA gene to identify microbes in the urine of children [5,9,10]. Although this modern, culture-independent method represents a clear advance, there are certain limitations as pointed out by Li et al. [1]. The authors concluded that culture-independent methods can only verify the presence of bacteria but fail to confirm their viability. Moreover, the different regions of the 16S-rRNA gene used may vary from study to study, thus making comparison of results difficult.

While our study revealed a significant difference between boys and girls with respect to the number of microbial strains occurring in combinations in the urine, there was no difference in terms of microbial diversity as such. However, *CoNS* and *Peptococci* predominated in the urine of boys, while *Enterococci* and *Eubacteria* prevailed in the urine of girls, with *Lactobacillus* strains detected in isolated cases. In 2020, Nelson et al. [20] verified 72 bacterial genera in the urine of healthy men, with the predominant microorganisms being *Corynebacteria*, *Streptococci*, and *Sneathia*. These findings in healthy men were confirmed by other authors [8,21]. Thus, certain species of the urinary microbiota in boys appear to constitute the core of the urinary microbiota of healthy male adults.

The presence of *Lactobacillus* in the urine of healthy women of reproductive age was described more than 10 years ago. The initial findings reported by Siddiqui et al. [22] were subsequently confirmed in several other studies [8,21,23]. Naboka et al. [24] hypothesized that *Lactobacilli* found rarely in the urine of prepubescent girls becomes dominant with increasing age, in line with the vaginal microbiota. Interestingly, the dominance of *Enterococci* in the urine of prepubescent girls is similar to that in postmenopausal women [24].

A few studies on the urinary microbiome in children have been published. By sequencing the 16S-rRNA gene (region V4) by Illumina MiSeg in the urine of healthy children, Fredsgaard et al. [5] showed that *Porphyromonas* spp. dominate in boys (22.4%). Other microorganisms detected in this study included *Ezakiella* (12.0%), *Campylobacter* (11.6%), *Prevotella* (8.6%), and *Dialister* (3.7%). *Prevotella* spp. prevailed in the urine of girls (18.2%), while *Porphyromonas* genera (12.9%), *Ezakiella* (8.1%), and *Dialister* (7.0%) were somewhat less prevalent. Kinneman et al. [10], who analyzed the urine of children with fever of unknown origin, reported *Prevotella*, *Peptoniphilus*, *Escherichia*, *Veillonella*, and *Finegoldia* as the most prevalent microorganisms. Kinneman et al. examined catheter urine samples in children (n = 85) below the age of 2 years who suffered from fever and suspected UTI [10]. The urine of children who had received antibiotics during the 2 weeks before urine collection exhibited lower α-diversity of microorganisms. However, the use of antibiotics did not significantly change Shannon and Simpson indices. The authors reported *Prevotella*, *Peptoniphilus*, *Escherichia*, *Veilonella*, and *Finegoldia* as the most prevalent microorganisms. The discrepancies compared to our findings may be explained by the specific cohort studied by Kinneman et al. [10].

In boys (mean age 7 months) undergoing circumcision, 12 identical types of urinary microorganisms (*Staphylococcus*, *Nocardiopsis*, *Acinetobacter*, *Pseudomonas*, *Corynebacterium*, *Nesterenkonia*, *Aliihoeflea*, *Saccharopolyspora*, *Sphingobacterium*, *Escherichia-Shigella*, *Lactobacillus*, and *Halomonas*) were isolated in 48 of 50 urine samples using 16S-rRNA amplicon sequencing [25]. This indicates a stable composition of the urinary microbiota in healthy boys. Diversity of the microbial spectrum did not appear to be affected by the method of delivery at birth. Similarly, Reasoner et al. studied the microbiome of urine taken by a catheter during circumcision in 50 healthy male infants (mean age 7 months) [26]. Using two methods (i.e., enhanced cultivation and sequencing of 16S-rRNA amplicons), the authors identified several common bacterial genera verified by either method. Using enhanced culture, *Nocardiopsis* and *Acinetobacter* were detected in all 50 urine samples, while *Staphylococcus*, *Escherichia*, *Shigella*, *Pseudomonas* and *Nocardiopsis* were isolated from >45 of 50 urine samples. The most common microorganisms cultured were *Escherichia*, *Shigella*, *Prevotella*, *Nocardiopsis*, *Lactobacter*, *Staphylococcus*, and *Lactobacillus*. The method of delivery at birth did not affect the composition of the urinary microbiota in these infants [26].

When comparing our results with those obtained in the two studies discussed above, the only microorganisms detected in all three studies were *Staphylococcus*, *Escherichia*, and *Lactobacillus*. The differences in composition may be due to divergent methods of bacterial analysis as well as the children’s age and other factors. The literature on the microbiota and/or microbiome of urine in girls is even more scarce, and therefore cannot be compared with our findings. Since the influence of gender as well as age on the urinary microbiota and/or microbiome of healthy children has been insufficiently well studied, further research is clearly warranted. Moreover, changes of the microbiota in the presence of UTI should be better understood to distinguish the normal microbiota from that in sick children.

## 5. Study Limitations and Strengths

Our pilot study included urine samples of 32 healthy prepubertal children. Due to the low number of participants, our study results must be interpretated with caution, and adequately powered prospective studies are required to confirm our results.

Because we investigated urine samples of girls and boys aged between 4 and 10 years, a possible influence of age on the final results cannot be excluded.

It should be noted that not all microorganisms can be cultured in commonly used culture media. Thus, we may have missed certain microorganisms in our analysis. Use of an expanded set of nutrient media would possibly reveal additional species of microorganisms in the urine. However, compared to the standard nutrient media, the nutrient media we used in this study were able to identify a wider range of aerobic and anaerobic species of microorganisms in urine. We hypothesize that 16S rRNA sequencing in combination with cultural techniques (using an expanded set of nutrient media) could complement our results on the urine microbiota of healthy children.

The results of this study are relevant to clinical practice by defining the standard microbiota of healthy prepubescent girls and boys.

## 6. Conclusions

The urine of healthy prepubescent children contained various aerobic–anaerobic combinations of microorganisms. Their diversity in the urine of girls and boys did not differ significantly. However, the level of α-diversity of microorganisms was higher in girls than in boys. We noted differences in the prevalence of certain taxa of microorganisms in the urine of boys and girls. Our study showed a close functional relationship between aerobic and anaerobic microorganisms detected in the urine of children in more than half of the cases.

## Figures and Tables

**Table 1:** Urinary microbiota in healthy children.

Microorganisms	Boys (n = 18)		Girls (n = 14)	
	Detection Rate (%)	Median Bacteriuria Level: Med [LQ; UQ], CFU/mL	Detection Rate (%)	Median Bacteriuria Level: Med [LQ; UQ], CFU/mL
*CoNS*	72.2	2 [2.00; 2.00]	50	2 [2.00; 2.00]
*S. lentus*	38.9	2 [2.00; 2.00]	21.4	2 [2.00; 2.00]
*S. haemolyticus*	27.8	2 [2.00; 2.00]	14.3	2 [2.00; 2.00]
*S. warneri*	11.1	2 [2.00; 2.00]	21.4	2 [2.00; 2.00]
*S. epidermidis*	11.1	2 [2.00; 2.00]	7.1	2 [2.00; 2.00]
*S. aureus*	5.6	2 [2.00; 2.00]	14.3	2 [2.00; 2.00]
*S. saprophyticus*	5.6	2 [2.00; 2.00]	7.1	2 [2.00; 2.00]
*Corynebacterium* spp.	38.9	2.43 [2.00; 3.00]	57.1	2.13 [2.00; 2.00]
*Enterococcus* spp.	38.9	2 [2.00; 2.00]	64.3	2 [2.00; 2.00]
*E. faecalis*	27.8	2 [2.00; 2.00]	28.6	2 [2.00; 2.00]
*E. faecium*	11.1	2 [2.00; 2.00]	0	0
*Enterococcus* undif.	11.1	2 [2.00; 2.00]	42.9 *	2 [2.00; 2.00]
*E. coli*	16.7	2 [2.00; 2.00]	35.7	2 [2.00; 2.00]
*C. tropicalis*	0	0	7.1	3 [3.00; 3.00]
*C. krusei*	0	0	7.1	3 [3.00; 3.00]
*Peptococcus* spp.	61.1	2 [2.00; 2.00]	71.4	2 [2.00; 2.00]
*Eubacterium* spp.	50	2.78 [2.00; 3.50]	78.6	2.18 [2.00; 2.00]
*Peptostreptococcus* spp.	38.9	2 [2.00; 2.00]	28.6	2.25 [2.00; 2.75]
*Propionibacterium* spp.	27.8	2 [2.00; 2.00]	57.1	2 [2.00; 2.00]
*Veillonella* spp.	16.7	2 [2.00; 2.00]	0	0
*Fusobacterium* spp.	5.6	2 [2.00; 2.00]	0	0
*Lactobacillus* spp.	5.6	2 [2.00; 2.00]	7.1	4 [4.00; 4.00]
*Prevotella* spp.	5.6	2 [2.00; 2.00]	0	0

* *p* ˂ 0.05; Med—median, LQ—lower quartile, UQ—upper quartile, CFU—colony-forming units, *CoNS*—coagulase-negative staphylococcus.

**Table 2:** α-Diversity of urinary microbiota in boys and girls (n = 32).

α-Diversity Values	Boys (n = 18)			Girls (n = 14)			*p* (Shapiro–Wilk Test)	*p* *
	Mean (±SD)	Min-Max	Median (Q25%; Q75%)	Mean (±SD)	Min-Max	Median (Q25%; Q75%)		
Number of species	5.44 (±2.18)	2.00–10.00	5.00 (4.00; 7.00)	6.71 (±3.17)	2.00–14.00	7.00 (3.75; 8.00)	0.103	0.213
Shannon index	1.26 (±0.56)	0.09–2.30	1.39 (0.92; 1.61)	1.51 (±0.45)	0.69–2.30	1.48 (1.14; 1.86)	0.274	0.168
Simpson index	0.35 (±0.18)	0.00–0.54	0.38 (0.24; 0.52)	0.40 (±0.13)	0.18–0.54	0.40 (0.29; 0.54)	0.002	0.295
Pielou’s evenness index	0.81 (±0.30)	0.05–1.00	1.00 (0.68; 1.00)	0.88 (±0.18)	0.44–1.00	1.00 (0.71; 1.00)	<0.001	0.710

* *t*-test of means’ equality (Welch modification) for normally distributed data; Mann–Whitney test for non-normally distributed data.

**Table 3:** Gender composition of clusters (n = 32 children).

Gender	Cluster		Total
	1 (n = 14)	2 (n = 18)	n = 32
Boys	10 (71.4%)	8 (44.4%)	18 (56.3%)
Girls	4 (28.6%)	10 (55.6%)	14 (43.8%)

**Table 4:** Heatmap of microbial biodiversity in urine of boys and girls. The values are highlighted in the appropriate color.

Microorganism	Boys (%)	Girls (%)	Microorganism
*CoNS*	72.2	78.6	*Eubacterium* spp.
*Peptococcus* spp.	61.1	71.4	*Peptococcus* spp.
*Eubacterium* spp.	50.0	64.3	*Enterococcus* spp.
*Corynebacterium* spp.	38.9	57.1	*Corynebacterium* spp.
*S. lentus*	38.9	57.1	*Propionibacterium* spp.
*Enterococcus* spp.	38.9	50.0	*CoNS*
*Peptostreptococcus* spp.	38.9	42.9	*Enterococcus undif.*
*S. haemolyticus*	27.8	35.7	*Enterobacteriaceae*
*E. faecalis*	27.8	35.7	*E. coli*
*Propionibacterium* spp.	27.8	28.6	*E. faecalis*
*Enterobacteriaceae*	16.7	28.6	*Peptostreptococcus* spp.
*E. coli*	16.7	21.4	*S. lentus*
*Veillonella* spp.	16.7	21.4	*S. warneri*
*S. warneri*	11.1	14.3	*S. haemolyticus*
*S. epidermidis*	11.1	14.3	*S. aureus*
*Enterococcus undif.*	11.1	14.3	*Candida* spp.
*E. faecium*	11.1	7.1	*S. saprophyticus*
*S. saprophyticus*	5.6	7.1	*S. epidermidis*
*S. aureus*	5.6	7.1	*Lactobacillus* spp.
*Lactobacillus* spp.	5.6	7.1	*C. tropicalis*
*Fusobacterium* spp.	5.6	7.1	*C. krusei*
*Prevotella* spp.	5.6	0	*E. faecium*
*Candida* spp.	0	0	*Fusobacterium* spp.
*C. tropicalis*	0	0	*Veillonella* spp.
*C. krusei*	0	0	*Prevotella* spp.

**Table 5:** Statistically significant relationships between detection rate of microorganisms in urine of boys and girls.

Microorganism	Boys (n = 18)	Girls (n = 14)
*Corynebacterium* spp.	–	*Propionibacterium* spp. C*_p_* = 0.578 (*p* = 0.026)
*S. haemolyticus*	*S. lentus* C*_p_* = 0.463 (*p* = 0.048)	*E. coli* C*_p_* = 0.480 (*p* = 0.040) *Lactobacillus* spp. C*_p_* = 0.562 (*p* = 0.011) *C. tropicalis* C*_p_* = 0.562 (*p* = 0.011)
*S. saprophyticus*	–	*S. lentus* C*_p_* = 0.469 (*p* = 0.047)
*S. lentus*	*Propionibacterium* spp. C*_p_* = 0.443 (*p* = 0.036) *Eubacterium* spp. C*_p_* = 0.495 (*p* = 0.016)	-
*S. warneri*	*Fusobacterium* spp. C*_p_* = 0.566 (*p* = 0.004)	*Lactobacillus* spp. C*_p_* = 0.469 (*p* = 0.047) *C. tropicalis* C*_p_* = 0.469 (*p* = 0.047)
*S. epidermidis*	*Lactobacillus* spp. C*_p_* = 0.566 (*p* = 0.004)	*S. aureus* C*_p_* = 0.562 (*p* = 0.011)
*S. aureus*	*Enterococcus undif.* C*_p_* = 0.566 (*p* = 0.004) *Veillonella* spp. C*_p_* = 0.477 (*p* = 0.021) *Prevotella* spp. C*_p_* = 0.707 (*p* < 0.001)	*Peptostreptococcus* spp. C*_p_* = 0.542 (*p* = 0.016) *S. epidermidis* C*_p_* = 0.562 (*p* = 0.011)
*E. faecalis*	*E*. *faecium* C*_p_* = 0.495 (*p* = 0.016) *Eubacterium* spp. C*_p_* = 0.527 (*p* = 0.031) *Peptococcus* spp. C*_p_* = 0.463 (*p* = 0.026) *Peptostreptococcus* spp. C*_p_* = 0.443 (*p* = 0.036)	*Candida* spp. C*_p_* = 0.542 (*p* = 0.016)
*E. faecium*	*Propionibacterium* spp. C*_p_* = 0.495 (*p* = 0.016) *E. faecalis* C*_p_* = 0.495 (*p* = 0.016)	-
*Lactobacillus* spp.	-	*C. tropicalis* C*_p_* = 0.707 (*p* < 0.001)
*Propionibacterium* spp.	*Peptostreptococcus* spp. C*_p_* = 0.443 (*p* = 0.036)	-
*Eubacterium* spp.	*Peptococcus* spp. C*_p_* = 0.495 (*p* = 0.016)	*C. krusei* C*_p_* = 0.469 (*p* = 0.047)
*Veillonella* spp.	*Prevotella* spp. C*_p_* = 0.477 (*p* = 0.021) *C. tropicalis* C*_p_* = 0.477 (*p* = 0.021)	–

Pearson’s contingency coefficients are shown, with significance levels calculated using Monte Carlo method (10,000 samples, 99% confidence level).

## Data Availability

Public access of data is restricted due to privacy restrictions of the institution. Data from this study can be obtained from the principal author on reasonable request.

## References

[1] Li J.K.M., Chiu P.K.F., Ng C.F. (2019). The impact of microbiome in urological diseases: A systematic review. Int. Urol. Nephrol..

[2] Kogan M.I., Naboka Y.L., Ibishev K.S., Gudima I.A., Naber K.G. (2015). Human urine is not sterile—Shift of paradigm. Urol. Int..

[3] Perez-Carrasco V., Soriano-Lerma A., Soriano M., Gutiérrez-Fernández J., Garcia-Salcedo J.A. (2021). Urinary microbiome: Yin and yang of the urinary tract. Front. Cell Infect. Microbiol..

[4] Wolfe A.J., Toh E., Shibata N., Rong R., Kenton K., FitzGerald M., Mueller E.R., Schreckenberger P., Dong Q., Nelson D.E. (2012). Evidence of uncultivated bacteria in the adult female bladder. J. Clin. Microbiol..

[5] Fredsgaard L., Thorsteinsson K., Bundgaard-Nielsen C., Ammitzbøll N., Leutscher P., Chai Q., Jensen A.M., Sørensen S., Pedersen L.M., Hagstrøm S. (2021). Description of the voided urinary microbiota in asymptomatic prepubertal children—A pilot study. J. Pediatr. Urol..

[6] Kawalec A., Zwolińska D. (2022). Emerging role of microbiome in the prevention of urinary tract infections in children. Int. J. Mol. Sci..

[7] Ackerman A.L., Chai T.C. (2019). The bladder is not sterile: An update on the urinary microbiome. Curr. Bladder Dysfunct. Rep..

[8] Fouts D.E., Pieper R., Szpakowski S., Pohl H., Knoblach S., Suh M.J., Huang S.T., Ljungberg I., Sprague B.M., Lucas S.K. (2012). Integrated next-generation sequencing of 16S rDNA and metaproteomics differentiate the healthy urine microbiome from asymptomatic bacteriuria in neuropathic bladder associated with spinal cord injury. J. Transl. Med..

[9] Kassiri B., Shrestha E., Kasprenski M., Antonescu C., Florea L.D., Sfanos K.S., Wang M.H. (2019). A Prospective study of the urinary and gastrointestinal microbiome in prepubertal males. Urology.

[10] Kinneman L., Zhu W., Wong W.S.W., Clemency N., Provenzano M., Vilboux T., Jane’t K., Seo-Mayer P., Levorson R., Kou M. (2020). Assessment of the urinary microbiome in children younger than 48 months. Pediatr. Infect. Dis. J..

[11] Gerber D., Forster C.S., Hsieh M. (2018). The role of the genitourinary microbiome in pediatric urology: A review. Curr. Urol. Rep..

[12] Forster C.S., Panchapakesan K., Stroud C., Banerjee P., Gordish-Dressman H., Hsieh M.H. (2020). A cross-sectional analysis of the urine microbiome of children with neuropathic bladders. J. Pediatr. Urol..

[13] Barr-Beare E., Saxena V., Hilt E.E., Thomas-White K., Schober M., Li B., Becknell B., Hains D.S., Wolfe A.J., Schwaderer A.L. (2015). the interaction between *Enterobacteriaceae* and calcium oxalate deposits. PLoS ONE.

[14] Kozlov R.S., Menshikov V.V., Mikhailov V.S., Shulyak B.F., Dolgikh T.I., Kruglov A.N., Alieva E.V., Malikova V.E. (2024). Clinical Recommendations “Bacteriological Analysis of Urine”. M. 2014, 33. Moscow, Russia. https://microbius.ru/uploads/document/file/28/klinicheskie-rekomendacii-bakteriologicheskij-analiz-mochi-moskva-2014.pdf.

[15] Wagner B.D., Grunwald G.K., Zerbe G.O., Mikulich-Gilbertson S.K., Robertson C.E., Zemanick E.T., Harris J.K. (2018). On the use of diversity measures in longitudinal sequencing studies of microbial communities. Front. Microbiol..

[16] Thomas-White K., Brady M., Wolfe A.J., Mueller E.R. (2016). The bladder is not sterile: History and current discoveries on the urinary microbiome. Curr. Bladder Dysfunct. Rep..

[17] Xu R., Deebel N., Casals R., Dutta R., Mirzazadeh M. (2021). A new gold rush: A review of current and developing diagnostic tools for urinary tract infections. Diagnostics.

[18] Robertson R.C., Manges A.R., Finlay B.B., Prendergast A.J. (2019). the human microbiome and child growth—First 1000 days and beyond. Trends Microbiol..

[19] Roswall J., Olsson L.M., Kovatcheva-Datchary P., Nilsson S., Tremaroli V., Simon M.C., Kiilerich P., Akrami R., Krämer M., Uhlén M. (2021). Developmental trajectory of the healthy human gut microbiota during the first 5 years of life. Cell Host Microbe.

[20] Nelson D.E., Van Der Pol B., Dong Q., Revanna K.V., Fan B., Easwaran S., Sodergren E., Weinstock G.M., Diao L., Fortenberry J.D. (2010). Characteristic male urine microbiomes associate with asymptomatic sexually transmitted infection. PLoS ONE.

[21] Whiteside S.A., Razvi H., Dave S., Reid G., Burton J.P. (2015). The microbiome of the urinary tract--a role beyond infection. Nat. Rev. Urol..

[22] Siddiqui H., Nederbragt A.J., Lagesen K., Jeansson S.L., Jakobsen K.S. (2011). Assessing diversity of the female urine microbiota by high throughput sequencing of 16S rDNA amplicons. BMC Microbiol..

[23] Pearce M.M., Hilt E.E., Rosenfeld A.B., Zilliox M.J., Thomas-White K., Fok C., Kliethermes S., Schreckenberger P.C., Brubaker L., Gai X. (2014). The female urinary microbiome: A comparison of women with and without urgency urinary incontinence. mBio.

[24] Naboka Y.L., Rymashevsky A.N., Kogan M.I., Gudima I.A., Borovleva O.A., Jalagonia K.T., Zarutskiy S.A. (2016). Microbiota of urine and vagina of healthy postmenopausal women (a pilot study). Urologiia.

[25] Hadjifrangiskou M., Reasoner S., Flores V., Van Horn G., Morales G., Peard L., Abelson B., Manuel C., Lee J., Baker B. (2023). Defining the infant male urobiome and moving towards mechanisms in urobiome research. Res. Sq..

[26] Reasoner S.A., Flores V., Van Horn G., Morales G., Peard L.M., Abelson B., Manuel C., Lee J., Baker B., Williams T. (2023). Survey of the infant male urobiome and genomic analysis of *Actinotignum* spp.. NPJ Biofilms Microbiomes.

